# PIGR predicts good clinical outcomes and plays a tumor suppressor role in the development of breast cancer

**DOI:** 10.3389/fonc.2025.1439120

**Published:** 2025-05-02

**Authors:** Tengfei Jiang, Zhi Sun, Ke Dong, Qian Pu, Haidong Gao, Yanrong Lv, Peng Li, Guanghui Zhao

**Affiliations:** ^1^ Medical Laboratory Center, Qilu Hospital of Shandong University (Qingdao), Qingdao, China; ^2^ Cheeloo College of Medicine, Shandong University, Jinan, China; ^3^ Department of Breast Diseases (II), Shandong Second Provincial General Hospital, Jinan, China; ^4^ Department of Breast Surgery, Qilu Hospital (Qingdao), Qingdao, China; ^5^ Department of Breast Surgery, General Surgery, Qilu Hospital of Shandong University, Jinan, China; ^6^ Department of Laboratory Medicine, Peking University People's Hospital, Qingdao, China; ^7^ Women and Children's Hospital, Qingdao University, Qingdao, China

**Keywords:** breast cancer, bioinformatics analysis, pIgR, prognosis, proliferation and metastasis

## Abstract

**Introduction:**

Breast cancer (BRCA) is a phenotypically and functionally heterogeneous disease. New biomarkers or therapeutic targets must be discovered to improve treatment effects. The polymeric immunoglobulin receptor (PIGR) plays an anti-cancer role in various human malignancies. This study aimed to explore the prognostic significance and possible function of PIGR in BRCA.

**Methods:**

Data from TCGA and GEO, and methods such as logistic regression analysis, Kaplan-Meier survival analysis, multivariate Cox analysis, GO, KEGG, and GSEA were employed to detect the effects of PIGR on BRCA bioinformatically. RT-qPCR, Western blot analysis, and immunohistochemistry were used to validate the expression of PIGR in BRCA. The effects of PIGR on BRCA were also detected *in vitro* and *in vivo*.

**Results:**

The expression level of PIGR was down-regulated in BRCA tissues. CCK-8 proliferation and colony formation assay demonstrated that overexpression of PIGR could inhibit breast cancer cell proliferation, clone formation, and migration. *In vivo* experiments confirmed these results. GSEA revealed that PIGR differentially expressed genes are mainly associated with the immune response. The expression level of PIGR significantly correlated with the infiltration of immune cells and the abundance of immune-related molecules.

**Conclusions:**

PIGR can suppress breast cancer cell growth *in vitro* and *in vivo* and is an independent protective factor for BRCA patients’ prognosis. PIGR correlates with tumor immunity and exerts anti-tumor effects in BRCA. PIGR might be a novel prognostic biomarker and therapeutic target.

## Introduction

The incidence of breast cancer (BRCA) continues to rise globally (1). BRCA has become the leading cause of female cancer globally in 2020, accounting for 1 in 4 cases and 1 in 6 cancer-related deaths worldwide (2). BRCA is a phenotypically and functionally heterogeneous disease (3). Prognostic indicators, including molecular classification, the Ki-67 proliferation index, pathological grading, and TNM staging, have exposed many shortcomings in clinical practice (4, 5). With the emergence and application of targeted molecular therapies, the prognosis of BRCA patients has continued to improve (6). Nevertheless, it is still not very satisfactory. Therefore, further research is necessary to identify effective biomarkers and therapeutic targets.

The polymeric immunoglobulin receptor (PIGR), a member of the immunoglobulin superfamily, facilitates the transcytosis of polymeric IgA and IgM across mucosal epithelial layers via endocytosis (7). By mediating this endocytic process, PIGR plays a vital role in mucosal immunity (8). At the cellular surface, the extracellular ligand-binding domain of PIGR is cleaved by proteolytic enzymes and released in a soluble form as secretory component (SC) or as part of secretory IgA (SIgA) into the lumen (9). Furthermore, PIGR acts as an important inflammatory mediator and is involved in the pathogenesis of liver fibrosis, pneumococcal meningitis, and chronic obstructive pulmonary disease (COPD) (10–12). Additionally, PIGR is recognized as a negative prognostic biomarker that may facilitate tumor progression in hepatocellular carcinoma and pancreatic ductal adenocarcinoma (13–17). Evidence also suggests that decreased PIGR expression correlates with tumor progression and poor prognosis in several cancers, including nasopharyngeal carcinoma, esophageal squamous cell carcinoma, gastric adenocarcinoma, lung cancer, and epithelial ovarian cancer (18–22). However, the relationship between BRCA and PIGR remains underexplored. This study aims to investigate the biological functions of PIGR in the context of BRCA.

The tumor microenvironment (TME) is populated by diverse immune cell types, which can be classified into anti-tumor and pro-tumor immune cells (23). Among these, T cells serve as the primary effectors of anti-tumor immune responses, predominantly comprising cytotoxic CD8^+^T cells and effector CD4^+^T cells. These T cells secrete cytokines such as IFN-γ and TNF-α, which induce cytotoxic effects on tumor cells (24). Additionally, natural killer (NK) cells, dendritic cells (DCs), and M1-polarized macrophages also contribute to anti-tumor immunity through the secretion of pro-inflammatory cytokines, including TNF-α, IL-6, IL-8, and IL-12 (25–27). In contrast, pro-tumor immune cells, primarily represented by regulatory T cells (Tregs), play a critical role in maintaining immune homeostasis and peripheral tolerance, thereby preventing excessive inflammation and limiting tissue damage (28). Within the TME, Tregs act as a feedback mechanism that suppresses anti-tumor immune responses. In light of the roles of these immune cell types, scientists have developed various immunotherapeutic strategies, including immune checkpoint inhibitors, CAR-T cell therapies, and DC-based vaccines, all aimed at reshaping immune function to enhance anti-tumor immunity (29). Notably, PIGR has been identified as playing an essential role in linking innate and adaptive immunity (9). This raises the hypothesis that targeting PIGR could represent a viable strategy for enhancing immunotherapeutic efficacy.

In this study, we apply bioinformatics methodologies to assess the prognostic implications of PIGR in BRCA and explore its potential as a target for immunotherapy. Through comprehensive analysis of publicly available datasets, we aim to elucidate the correlation between PIGR expression and clinical outcomes in BRCA patients. To further substantiate our findings, we validate the biological functions of PIGR in both *in vitro* and *in vivo* models. This research seeks to identify novel therapeutic strategies that could enhance clinical management and improve prognoses for BRCA patients.

## Materials and methods

### Data source and online analysis tool

We acquired samples with sufficient clinical information from the Cancer Genome Atlas (TCGA) database ( *https://portal.gdc.cancer.gov/* ). We also downloaded data samples from the GSE10810 and GSE86166 datasets of the Gene Expression Omnibus (GEO) database ( *https://www.ncbi.nlm.nih.gov/geo/* ). R (3.6.3 version) with stats (4.2.1 version), ggplot2 (3.3.6 version), car (3.1.0 version), and pROC (1.18.0 version) were utilized to process the original data. Other online tools used in this study include TISIDB ( *http://cis.hku.hk/TISIDB/index.php* ), Kaplan-Meier Plotter ( *http://kmplot.com/analysis/* ), UALCAN ( *http://ualcan.path.uab.edu/* ), GEPIA2 (http://gepia2.cancer-pku.cn/), and MethSurv (https://biit.cs.ut.ee/methsurv/).

### Differentially expression

We downloaded data from the TCGA database and performed logistic regression analysis to assess the correlation between PIGR expression and clinicopathological characteristics in BRCA. The Wilcoxon rank-sum test was used to compare PIGR expression levels between tumor tissues and corresponding normal tissues across 24 human malignancies. Subsequently, we analyzed PIGR expression in the TCGA-BRCA dataset and further validated these results using the GSE10810 dataset. Finally, a ROC curve was generated based on data from the TCGA-BRCA dataset to evaluate the accuracy of PIGR in identifying breast cancer tissues.

### Prognostic value

The log-rank test was used to assess the relationship between PIGR expression and the prognosis of BRCA patients. We utilized TISIDB to explore the associations between PIGR and clinical outcomes across 30 human malignancies. Kaplan-Meier curves for overall survival (OS) or recurrence-free survival (RFS) were generated based on data from the GSE86166 dataset (group cutoff value: 0.94) and the Kaplan-Meier plotter database, using the log-rank test for comparison. Prognostic factors for BRCA were presented in a forest plot derived from multivariate Cox regression analysis. Factors with a hazard ratio (HR) >1 and p-value <0.05 were considered risk factors, while those with HR <1 and p-value <0.05 were considered protective factors. A nomogram was constructed to predict the probability of 1-, 3-, and 5-year OS for BRCA patients. Calibration curves were generated to evaluate the prediction accuracy of the nomogram.

### Pathological samples collection and immunohistochemistry staining

A total of 18 paraffin-embedded BRCA tissue samples and their corresponding paracancerous normal tissues were obtained from the Pathology Department of Qilu Hospital (Qingdao). Informed consent was obtained from all patients or their families. IHC staining was conducted according to the manufacturer’s instructions. Tissues were fixed in 10% formalin, embedded in paraffin, sectioned at a thickness of 4-6 μm, and mounted on slides. Following deparaffinization, rehydration, and microwave-based antigen retrieval, the slides were incubated with Anti-PIGR (Rabbit, Cat#HPA012012, Sigma-Aldrich), Anti-CD3 polyclonal antibody(Rabbit, Ca#17617-1-AP, proteintech ), and Anti- CD20 polyclonal antibody( Rabbit, Ca #24828-1-AP, proteintech) at a 1:500 dilution at 4°C overnight. The slides were incubated with secondary antibody at room temperature for 30 min and stained with DAB substrate, followed by hematoxylin counterstaining.

### Cell culture and transfection

We observed the proliferation, clone formation, and migration of MCF-7, MDA‐MB‐231, and SKBR3 breast cancer cells in PIGR knockdown, overexpression, and the control groups. The cell lines were derived from the preservation in our laboratory. MCF-7 and MDA‐MB‐231 cell lines were cultured in Dulbecco’s Modified Eagle’s medium, and SKBR3 cells were cultured in McCoy’s 5A Medium. Both media were supplemented with 10% fetal bovine serum and 1% penicillin-streptomycin. Lentiviral vectors overexpressing PIGR (Lv-PIGR) and knockdown PIGR (shPIGR) were constructed, along with corresponding controls (Lv-CTRLand shCTRL), and confirmed by Genechen Co. (Shanghai, China). MCF-7 cells were infected with Lv-PIGR, shPIGR, and their respective control lentiviral vectors and selected with puromycin for two weeks to establish stable PIGR overexpressing or knockdown cell lines. For MDA-MB-231 and SKBR3 cell lines, PIGR overexpression plasmid(OE-PIGR)and empty vector (OE-CTRL) were transfected into the corresponding cell lines using Lipofectamine 3000 transfection reagent(Ca#L3000015,Thermo Fisher Scientific,)according to the manufacturer’s instructions. After 48 hours of incubation, proteins were extracted for verification.

### Cell counting kit 8 proliferation assay and clone formation assay

We seeded cells in 96-well plates following 4×10^3^ cells/well concentrations. We repeated the seeding thrice and added 100ul CCK8 reagent (CK04, Dojindo CO. Ltd., Kumamoto, Japan) to each well every 24 hours. After incubating for 1–4 hours, the absorbance at 450nm was measured by a microplate reader. To study cell clone formation, we seeded constructed cell lines in six-well plates at a concentration of 1×10^3^ cells per well and cultured them for two weeks. At the end of the culture period, the cells were washed three times with PBS. Subsequently, the cells were fixed with 4% paraformaldehyde for 15 minutes at room temperature. The fixed cells were stained with 0.5% crystal violet solution for 10–15 minutes. After staining, cells were washed thoroughly with PBS to remove excess dye. We used ImageJ software (version 1.51; National Institute of Health) for automated colony counting to ensure accuracy and consistency. Each experimental group and control group included at least three replicates.

### Transwell migration assay

We conducted a Transwell migration assay using a system comprising 24-well plates with 6.5 mm diameter inserts and 8.0μm pore size polycarbonate membrane (Corning Incorporated costar, Kennebunk, ME). According to the manufacturer’s protocol, 4.0×10^4^ cells were resuspended in 200μL of serum-free DMEM and seeded into the upper chambers of the Transwell inserts. A medium containing 20% FBS was added to the lower chambers. After 24 hours of incubation, non-migratory cells on the upper surface of the membrane were removed with a cotton swab. Migratory cells on the lower surface were fixed with methanol and stained with crystal violet. The number of migrated cells was then quantified under a light microscope.

### RT-qPCR

Total RNA was extracted from cultured breast cancer cells using TRIzol reagent (Thermo Fisher Scientific, USA). The isolated RNA was then reverse transcribed into cDNA using the SynScript ^®^ III One-Tube RT SuperMix (+gDNA Remover) (Tsingke, Beijing, China) for subsequent analyses. Quantitative PCR (qPCR) was performed using the 1.1× EasyQ SYBR qPCR Mix (Low ROX Premixed) kit (Tsingke, Beijing, China). The primers were synthesized by Tsingke Biotechnology Co., Ltd. (Beijing, China). The sequences of the primers were: GAPDH, Forward GACTTCAACAGCG-3’, Reverse 5’-ACCACCCTGTTGCTGTAGCCAA-3’; PIGR, Forward 5’-TACTGGTGTGGAGTGAAGCAGG-3’, Reverse 5’- AGCACCTTCTCATCAGGAGCA

G-3’; HLA-A, Forward 5’-AGATACACCTGCCATGTGCAGC-3’, Reverse 5’- GATCACAGCTCC

AAGGAGAACC-3’; HLA-B,Forward 5’-CTGCTGTGATGTGTAGGAGGAAG-3’, Reverse 5’-GC

TGTGAGAGACACATCAGAGC-3’; HAVCR2, Forward 5’-GACTCTAGCAGACAGTGGGATC-3’, Reverse5’-GGTGGTAAGCATCCTTGGAAAGG-3’; CD274, Forward 5’-TGCCGACTACAAGC

GAATTACTG-3’, Reverse 5’-CTGCTTGTCCAGATGACTTCGG-3’. The quantitative real-time PCR (qPCR) experiments were conducted using the Applied Biosystems™ 7500 Real-Time PCR System (Thermo Fisher Scientific, USA). Relative quantification of gene expression was performed using the 2^(-ΔΔCt) method, with β-actin serving as the endogenous reference gene for normalization.

### Western blotting

Cells were washed twice with cold Phosphate Buffered Saline (PBS) and then lysed with Radio Immunoprecipitation Assay buffer (RIPA) (Cat#R0010; Beyotime Institute of Biotechnology) supplemented with PMSF on ice for 30 min. The lysate was centrifuged at 12,000 ×g at 4°C for 15 min. Total proteins were quantified using a bicinchoninic acid assay kit (Cat#PC0020; Solarbio). 20 μg protein were separated on a 10% sodium dodecyl-sulfate polyacrylamide gel and then transferred to a polyvinylidene difluoride (PVDF) membrane. The membrane was blocked with 5% skim milk at room temperature for 2 hours and then incubated with anti-β-actin (1:10000, Cat#66009-1-Ig; Proteintech, China) and anti-PIGR (1:5000, Cat#HPA012012; Atlas Antibodies, Sweden), overnight at 4°C. After washing three times with Tris Buffered Saline with Tween 20 (TBST), the membrane was incubated with secondary antibodies: Goat Anti-Rabbit IgG (H+L) horseradish peroxidase (1:5000, Cat# S0001, Affinity) or Goat Anti-Mouse IgG (H+L) horseradish peroxidase (1:5000, Cat#S0002, Affinity) for 1hour at room temperature. The target protein bands were visualized using an enhanced chemiluminescence kit (Cat# KF003, Affinity), and images were captured by a chemiluminescence system (Amersham Imager 600).

### *In vivo* assays

Female nude mice (4 to 6 weeks of age) were purchased from Beijing Vital River Laboratory Animal Technology Co., Ltd. (Beijing, China). All the animal experiments have been approved by the Medical Ethics Committee of Qilu Hospital (Qingdao) of Shandong University. The mice were randomized into two groups (five mice per group) before tumor cell inoculation. 5×10^6^ MCF-7 cells transduced with Lv-CTRL or Lv-PIGR were resuspended in 100 μL PBS and subcutaneously injected into the mice. Measurements were taken twice weekly starting from day 7. Tumor volumes were calculated using the standard formula: tumor volume = (L × W^2^) ×6/π. On day 28, the naked mice were euthanized, and tumor weights were measured.

### DNA methylation analysis

To explore the possible mechanisms underlying PIGR low expression in BRCA, we analyzed the differences in DNA promoter methylation levels between BRCA and normal breast tissues using the UALCAN website. We also examined the DNA methylation status of the PIGR gene and assessed the prognostic value of CpG island methylation status using the MethSurv database.

### Differential gene expression and functional enrichment analysis

Based on the median expression level of PIGR, breast cancer patients from the TCGA database were categorized into two groups: high and low PIGR expressers. Differential expression gene (DEG) analysis was conducted using the R package DESeq2 and edgeR (30, 31), with an adjusted p-value of less than 0.05 and a threshold of |log2-fold-change (FC)| >1 for identifying DEGs. The correlation of the top 10 DEGs with PIGR was assessed using Spearman’s rank correlation analysis.

Functional enrichment analyses, encompassing Gene Ontology (GO) and Kyoto Encyclopedia of Genes and Genomes (KEGG), were conducted on the differentially expressed genes (DEGs) utilizing the R package GOplot (version 1.0.2) as described by Walter et al. (32). Additionally, Gene Set Enrichment Analysis (GSEA) was performed employing the R package clusterProfiler (33, 34). Enriched functional or pathway terms were considered statistically significant with an adjusted p-value threshold of less than 0.05 and a false discovery rate (FDR) below 0.25.

### Immune cell infiltration analysis and immune-related signatures analysis

We investigated the relationship between PIGR expression and the infiltration levels of 24 immune cell types in BRCA using data from TCGA. Additionally, we explored the impact of PIGR expression on the infiltration of seven typical immune cells. Data processing was performed using R (3.6.3 version). We further examined the relationship between PIGR expression and the abundance of various immune molecules across multiple cancers using the TISIDB data platform. These immune molecules include tumor-infiltrating lymphocytes (TILs), immunomodulators (such as immune inhibitors, immunostimulators, and major histocompatibility complex (MHC) molecules), chemokines, and receptors.

### Statistical analysis

Statistical analyses were performed with PRISM version 9 (GraphPad Software Inc., San Diego, CA, USA) or R version 3.6.3. The differences between the groups were compared using Student’s t-test or one-factor analysis of variance (ANOVA). Statistically significant were shown as *p<0.05, **p<0.01, ***p<0.001, ****p<0.0001, and ns represents no significance. All results were repeated triplicate independently.

## Results

### PIGR expression patterns and their clinicopathological significance in BRCA

Through an in-depth analysis of data from 1083 BRCA patients in the TCGA database, we revealed detailed clinicopathological features associated with PIGR expression (**Table 1**). We further compared the characteristics of the high and low PIGR expression groups (**Table 2**). Significant associations were found between PIGR expression and several clinicopathological features, including age, histological type, progesterone receptor (PR) status, estrogen receptor (ER) status, PAM50 gene panel classification, and menopausal status in BRCA patients (**Table 3**).

**Table 1:** Clinicopathological characteristics of BRCA patients based on TCGA.

Characteristic	Levels	Overall
N		1083
T stage, n (%)	T1	277 (25.6%)
	T2	629 (58.2%)
	T3	139 (12.9%)
	T4	35 (3.2%)
N stage, n (%)	N0	514 (48.3%)
	N1	358 (33.6%)
	N2	116 (10.9%)
	N3	76 (7.1%)
M stage, n (%)	M0	902 (97.8%)
	M1	20 (2.2%)
Pathologic stage, n (%)	Stage I	181 (17.1%)
	Stage II	619 (58.4%)
	Stage III	242 (22.8%)
	Stage IV	18 (1.7%)
Race, n (%)	Asian	60 (6%)
	Black or African American	181 (18.2%)
	White	753 (75.8%)
Age, n (%)	<=60	601 (55.5%)
	>60	482 (44.5%)
Histological type, n (%)	Infiltrating Ductal Carcinoma	772 (79%)
	Infiltrating Lobular Carcinoma	205 (21%)
PAM50, n (%)	Normal	40 (3.7%)
	Luminal A	562 (51.9%)
	Luminal B	204 (18.8%)
	Her2	82 (7.6%)
	Basal	195 (18%)
Menopause status, n (%)	Pre	229 (23.6%)
	Peri	40 (4.1%)
	Post	703 (72.3%)
Anatomic neoplasm subdivisions, n (%)	Left	563 (52%)
	Right	520 (48%)
Age, median (IQR)		58 (48.5, 67)

**Table 2:** Clinicopathological characteristics of high- and low-PIGR expression groups in BRCA.

Characteristic	Levels	Low expression of PIGR	High expression of PIGR	p-value
N		541	542	
Age, median (IQR)		60 (50, 69)	57 (47, 65)	0.007
Age, n (%)	<=60	280 (25.9%)	321 (29.6%)	0.016
	>60	261 (24.1%)	221 (20.4%)	
T stage, n (%)	T1	128 (11.9%)	149 (13.8%)	0.050
	T2	329 (30.5%)	300 (27.8%)	
	T3	60 (5.6%)	79 (7.3%)	
	T4	22 (2%)	13 (1.2%)	
N stage, n (%)	N0	263 (24.7%)	251 (23.6%)	0.086
	N1	178 (16.7%)	180 (16.9%)	
	N2	59 (5.5%)	57 (5.4%)	
	N3	27 (2.5%)	49 (4.6%)	
M stage, n (%)	M0	459 (49.8%)	443 (48%)	0.238
	M1	7 (0.8%)	13 (1.4%)	
Pathologic stage, n (%)	Stage I	84 (7.9%)	97 (9.2%)	0.030
	Stage II	329 (31%)	290 (27.4%)	
	Stage III	110 (10.4%)	132 (12.5%)	
	Stage IV	5 (0.5%)	13 (1.2%)	
Race, n (%)	Asian	38 (3.8%)	22 (2.2%)	0.036
	Black or African American	96 (9.7%)	85 (8.6%)	
	White	350 (35.9%)	387 (39.7%)	
Histological type, n (%)	Infiltrating Ductal Carcinoma	410 (42%)	362 (37.1%)	< 0.001
	Infiltrating Lobular Carcinoma	63 (6.4%)	142 (14.5%)	
PR status, n (%)	Negative	199 (19.2%)	143 (13.8%)	< 0.001
	Indeterminate	1 (0.1%)	3 (0.3%)	
	Positive	314 (30.4%)	374 (36.2%)	
ER status, n (%)	Negative	145 (14%)	95 (9.2%)	< 0.001
	Indeterminate	0 (0%)	2 (0.2%)	
	Positive	370 (35.7%)	423 (40.9%)	
HER2 status, n (%)	Negative	275 (37.8%)	283 (38.9%)	0.341
	Indeterminate	8 (1.1%)	4 (0.6%)	
	Positive	84 (11.6%)	73 (10%)	
PAM50, n (%)	Normal	6 (0.6%)	34 (3.1%)	< 0.001
	Luminal A	237 (21.9%)	325 (30%)	
	Luminal B	132 (12.2%)	72 (6.6%)	
	Her2	40 (3.7%)	42 (3.9%)	
	Basal	126 (11.6%)	69 (6.4%)	
Menopause status, n (%)	Pre	100 (10.3%)	129 (13.3%)	0.047
	Peri	17 (1.7%)	23 (2.4%)	
	Post	368 (37.9%)	335 (34.5%)	
Anatomic neoplasm subdivisions, n (%)	Left	284 (26.2%)	279 (25.8%)	0.783
	Right	257 (23.7%)	263 (24.3%)	
Radiation therapy, n (%)	No	223 (22.6%)	211 (21.4%)	0.214
	Yes	261 (26.4%)	292 (29.6%)	
OS event, n (%)	Alive	456 (42.1%)	475 (43.9%)	0.134
	Dead	85 (7.8%)	67 (6.2%)	
DSS event, n (%)	Alive	483 (45.4%)	495 (46.6%)	0.606
	Dead	45 (4.2%)	40 (3.8%)	

HER2, human epidermal growth factor receptor 2; OS, overall survival; DSS, disease-specific survival.

**Table 3:** Logistics analysis of the correlation between PIGR expression and clinical characteristics in BRCA.

Clinical Characteristics	Total(N)	Odds Ratio(OR)	p-value
Age (>60 vs. <=60)	1,083	0.761 (0.598-0.968)	0.026
Race (Black or African American&White vs. Asian)	994	1.701 (1.002-2.944)	0.052
Menopause status (Post vs. Pre&Peri)	972	0.690 (0.519-0.915)	0.010
T stage (T2&T3&T4 vs. T1)	1,080	0.773 (0.587-1.016)	0.065
N stage (N1&N2&N3 vs. N0)	1,064	1.110 (0.873-1.412)	0.395
M stage (M1 vs. M0)	922	1.907 (0.774-5.116)	0.173
Pathologic stage (Stage III&Stage IV vs. Stage I&Stage II)	1,060	1.374 (1.037-1.822)	0.027
Histological type (Infiltrating Lobular Carcinoma vs. Infiltrating Ductal Carcinoma)	977	2.566 (1.855-3.583)	<0.001
ER status (Positive vs. Negative&Indeterminate)	1,035	1.599 (1.196-2.144)	0.002
PR status (Positive vs. Negative&Indeterminate)	1,034	1.520 (1.173-1.974)	0.002
HER2 status (Positive vs. Negative&Indeterminate)	727	0.915 (0.642-1.302)	0.621
Anatomic neoplasm subdivisions (Right vs. Left)	1,083	1.011 (0.797-1.284)	0.926
Radiation therapy (Yes vs. No)	987	1.231 (0.957-1.584)	0.106
PAM50 (Basal vs. Her2&Luminal B&Luminal A&Normal)	1,083	0.507 (0.367-0.696)	<0.001

**Figure 1:**
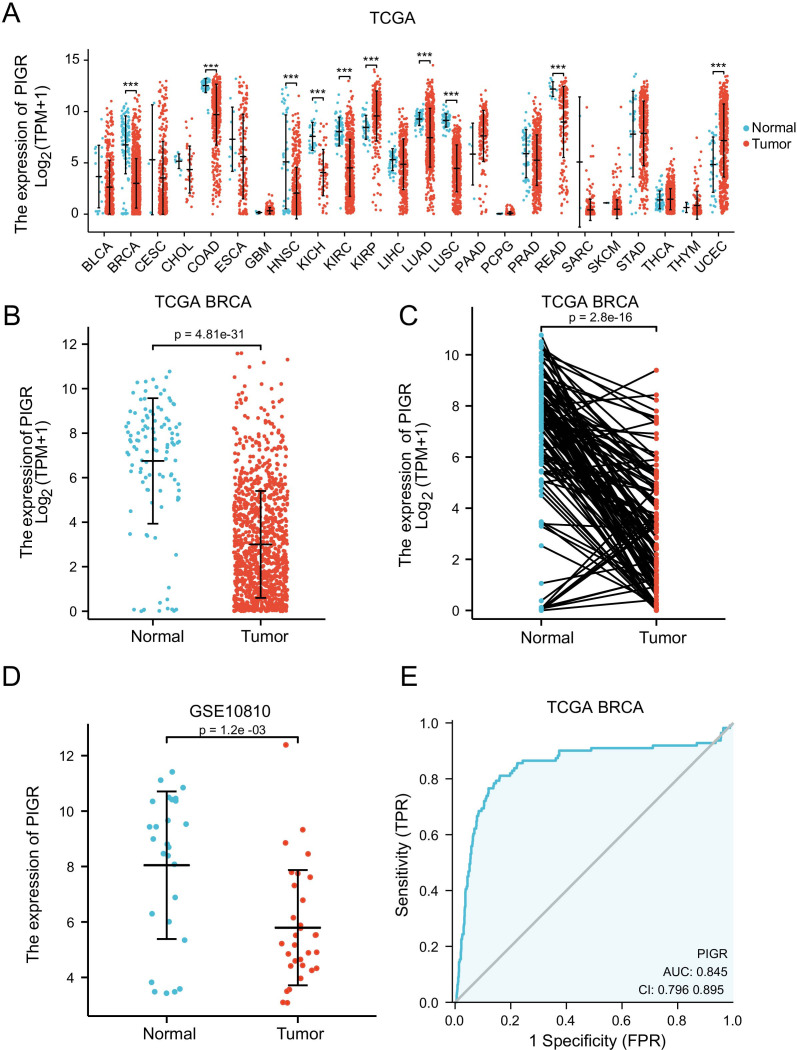
Expression levels of PIGR in 24 types of human malignancies, including BRCA. **(A)** Expression of PIGR in tissues of 24 types of human malignancies and corresponding normal tissues in the TCGA database. **(B)** Expression of PIGR in BRCA tissues and non-matched adjacent normal breast tissues in TCGA database (P ≤ 0.01). **(C)** Expression of PIGR in BRCA tissues and matched adjacent normal breast tissues in TCGA database (P ≤ 0.01). **(D)** Expression of PIGR in 31 BRCA tissues and 27 control samples in the GSE10810 dataset. **(E)** A ROC curve to verify the capacity of PIGR to discriminate between BRCA and normal breast tissues. (Abbreviations: TCGA, the cancer genome atlas; ROC, receiver operating characteristic; AUC, the area under the curve; TPR, true positive rate; FPR, false positive rate; CI, confidence interval. P-value Significant Codes: 0 ≤***< 0.001 ≤**< 0.01 ≤*< 0.05, ns, not significant).

**Figure 2:**
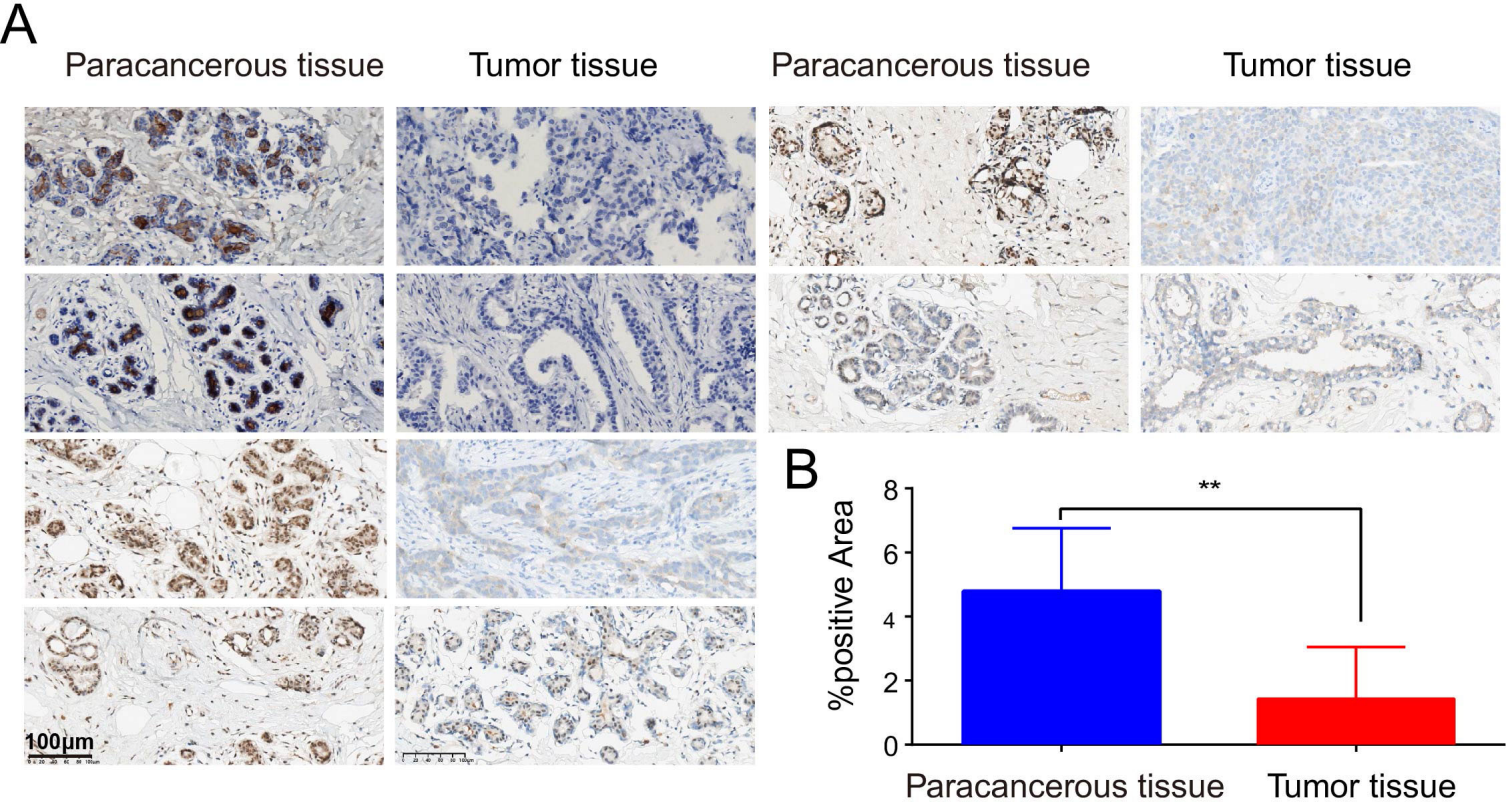
Detect of differential expression of PIGR in BRCA tissues and matched normal breast tissues by immunohistochemistry. **(A)** Six representative patients. **(B)** The proportion of PIGR staining positive areas in para-cancerous tissues and cancerous tissues.

In the pan-cancer expression analysis, we observed that PIGR expression levels were significantly lower in 8 out of 24 cancer types compared to corresponding normal tissues. Conversely, PIGR expression was significantly higher in 2 cancer types (**Figure 1A**). In the TCGA-BRCA dataset, PIGR expression was significantly reduced in both unpaired samples (**Figure 1B**) and paired samples (**Figure 1C**). These findings were corroborated by the GSE10810 dataset, which similarly demonstrated decreased PIGR expression in breast cancer tissues (**Figure 1D**). To further substantiate these findings, we employed IHC staining to evaluate PIGR expression in breast cancer tissues and corresponding normal tissues from 18 breast cancer patients. The results showed that PIGR expression was significantly downregulated in breast cancer tissues compared to adjacent normal tissues (**Figures 2A, B**). Additionally, receiver operating characteristics (ROC) curve analysis revealed that PIGR had considerable accuracy in distinguishing BRCA tissues from normal tissues, with an area under the curve (AUC) of 0.845 (**Figure 1E**).

### PIGR overexpression associated with favorable prognosis and effective long-term survival prediction in BRCA

**Figure 3:**
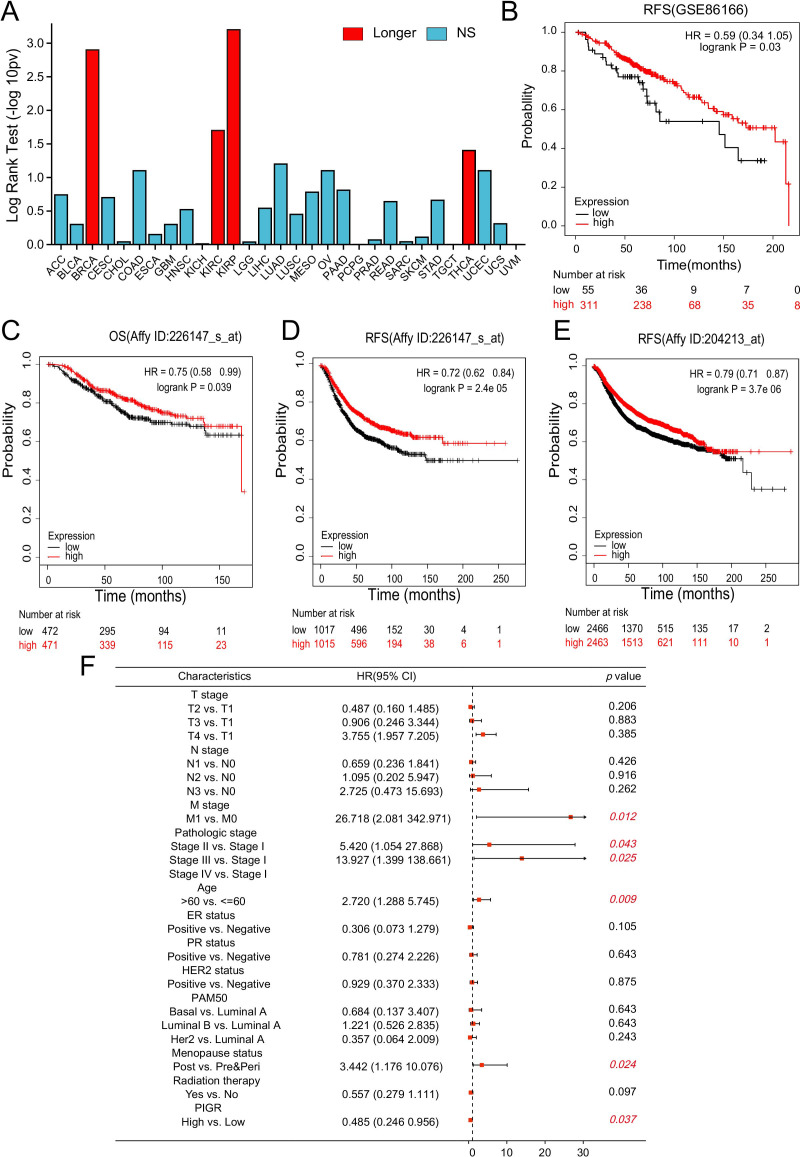
PIGR expression associated with favorable prognosis in BRCA. **(A)** The association between PIGR expression and OS in 30 types of human malignancies was based on data from the TISIDB. **(B)** Kaplan-Meier curves for RFS of BRCA patients with high and low PIGR expression based on data from the GSE86166 dataset. **(C-E)** Kaplan-Meier curves for OS or RFS of BRCA patients with high and low PIGR expression based on gene chip data from the Kaplan-Meier Plotter. **(F)** Forest map based on multivariate Cox analysis of PIGR and clinical parameters for OS. (OS, overall survival; RFS, relapse-free survival; HR, hazard ratio. Longer (or shorter), the gene is associated with longer (or shorter) OS (Log-rank test: P<0.05); NS, Not Significant).

Whether the expression of PIGR affects the prognosis of patients is of significant concern. Our analysis of the TISIDB database demonstrated that increased PIGR expression is significantly associated with improved OS in BRCA patients, with its prognostic value ranking second highest among 30 human malignancies (**Figure 3A**). This association was further supported by the GSE86166 dataset, which showed that BRCA patients with higher PIGR expression levels had better relapse-free survival (RFS) (**Figure 3B**). Consistent with these findings, data from the Kaplan-Meier plotter indicated that PIGR expression was significantly associated with better OS and RFS in BRCA patients (**Figures 3C-E**). Multivariate Cox regression analysis revealed that while higher M stage, higher pathologic stage, older age, and post-menopausal status were independent risk factors for OS in BRCA patients, higher PIGR expression was identified as an independent protective factor (**Figure 3F**).

Building on these findings, we developed a nomogram that integrates PIGR expression with other prognostic factors to predict the 1-, 3-, and 5-year OS probabilities for BRCA patients (**Supplementary Figure 4A**). This nomogram, which assigns higher total points for a worse prognosis, has been evaluated for its predictive accuracy by constructing calibration curves for 1-, 3-, and 5-year predictions (**Supplementary Figure 4B–D**). The nomogram exhibited moderate predictive accuracy for OS in BRCA patients, with a concordance index (C-index) of 0.701 (95% CI = 0.669-0.732), indicating its potential utility as a clinical tool for prognosis assessment.

### PIGR can suppress breast cancer cell growth *in vitro* and *in vivo*

**Figure 4:**
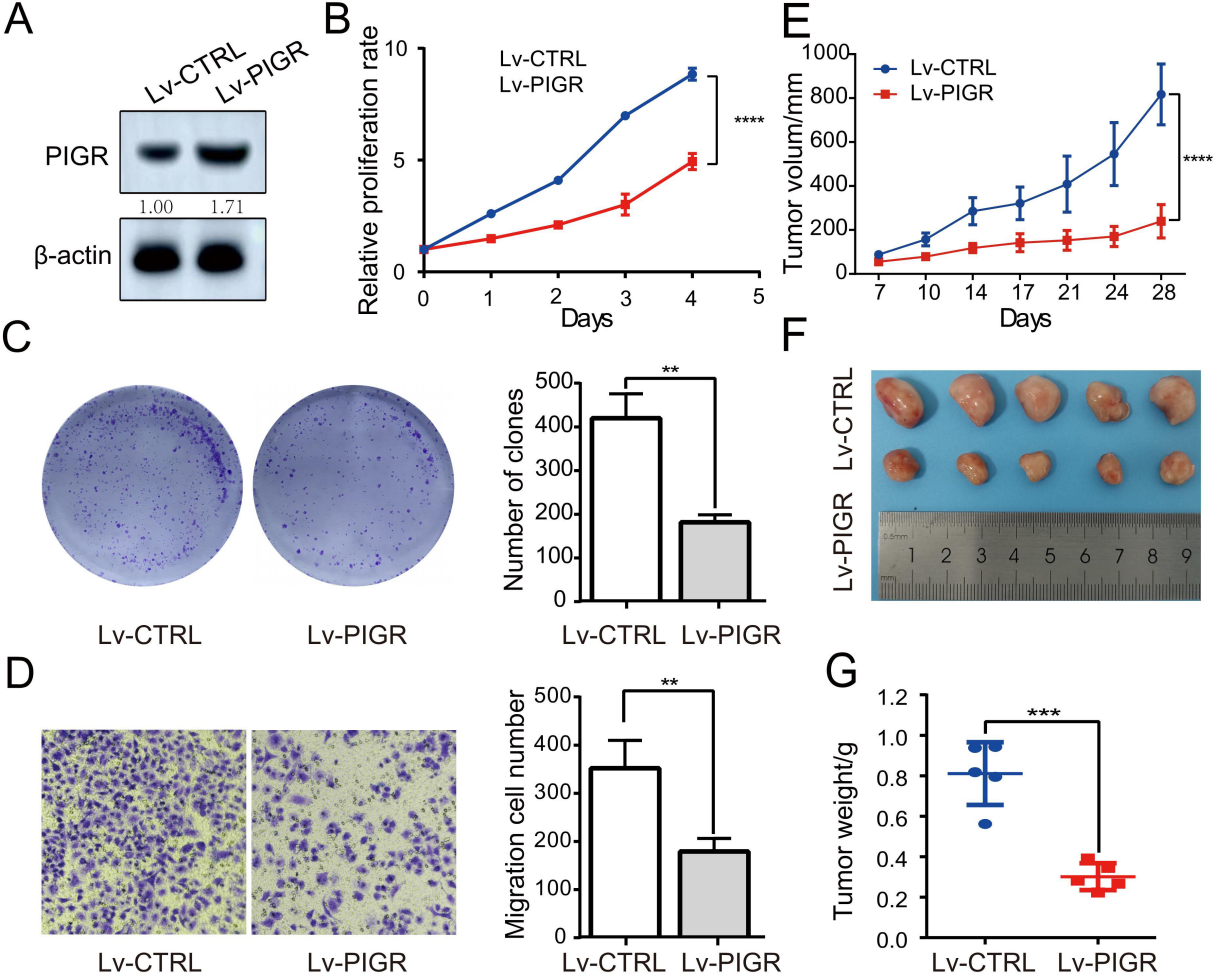
PIGR Can Suppress Breast Cancer Cell growth *in vitro* and *in vivo.* **(A)** Western blotting was used to test the expression of PIGR in PIGR overexpressing MCF-7 cells. **(B)** Effects of PIGR on MCF-7 cell proliferation checked via the CCK-8 assay. **(C)** Effects of PIGR on MCF-7 cell clone formation checked via a colony formation assay. **(D)** Effects of PIGR on MCF-7 migration checked via the Transwell migration assay. **(E)** xenograft tumor volumes in Lv-CTRL and Lv-PIGR groups on the indicated days. **(F-G)** Representative images and tumor weights in Lv-CTRL and Lv-PIGR groups on day 28. Each set of experiments includes five mice. * p<0.05. (Lv-CTRL, MCF-7 cells transfected with Lentiviral vectors as control; Lv-PIGR, MCF-7 cells transfected with Lentiviral vectors to overexpressing PIGR).

Given that PIGR plays an anti-cancer role in a variety of human malignancies, the above analysis prompted us to explore whether its high expression in breast cancer is related to the biological function of breast cancer cells. We confirmed the establishment of MCF-7 breast cancer cell lines with stably upregulated PIGR expression through Western blotting (**Figure 4A**). After 96 hours of incubation, the proliferation of cells in the Lv-PIGR group was significantly inhibited compared with the Lv-CTRL group (**Figure 4B**). The colony formation assay also showed that after 7 days of incubation with the medium, the proliferation of breast cancer cells in the Lv-PIGR group was significantly lower than that in the Lv-CTRL group (**Figure 4C**). Additionally, the results of the Transwell assay demonstrated that PIGR overexpression inhibited the migration ability of MCF-7 cells (**Figure 4D**). We also upregulated PIGR expression in SKBR3 and MDAMB-231 cell lines (**Supplementary Figure 1A**), both of which significantly inhibited cell proliferation (**Supplementary Figure 1B**), clonal formation (**Supplementary Figure 1C**), and migration (**Supplementary Figure 1D**).

We also generated a MCF-7 cell line with stable knockdown of PIGR, which was verified by Western blotting(**Supplementary Figure 2A**). We found that there were no statistically significant differences between the PIGR low expression group and the control group in either proliferation (**Supplementary Figure 2B**) or clone formation (**Supplementary Figure 2C**). Nevertheless, our Transwell migration assay showed significantly increased MCF-7 cell migration in the PIGR low expression group (**Supplementary Figure 2D**).

We extended our investigation to assess the impact of PIGR on tumorigenicity *in vivo* using a xenograft mouse model. MCF-7 cells stably transfected with either Lv-CTRL or Lv-PIGR were subcutaneously injected into nude mice (five mice per group). Tumor volume was monitored biweekly. On day 28, tumors from the Lv-PIGR group were significantly smaller compared to the Lv-CTRL group (**Figure 4E**). At the experimental endpoint on day 28, the mice were humanely euthanized, and tumors were harvested to measure size and weight. Consistent with the volume measurements, tumors from the Lv-PIGR group were both smaller and lighter than those from the Lv-CTRL group (**Figure 4F–G**). These results suggest that PIGR overexpression may inhibit tumor growth *in vivo*.

Furthermore, to explore the epigenetic regulation of PIGR, we utilized the UALCAN website to analyze DNA promoter methylation. Our analysis indicated that the methylation level of the PIGR promoter was significantly higher in primary breast cancer (BRCA) tissues compared to normal breast tissues (**Supplementary Figure 3A**). Hypermethylation was observed in most of the CpG islands within the PIGR DNA sequences in BRCA tissues (**Supplementary Figure 3B**). Notably, hypermethylation of two out of eight CpG islands was associated with improved overall survival (OS) in BRCA patients (**Table 4**), suggesting a potential link between PIGR promoter methylation and BRCA prognosis.

**Table 4:** Effects of methylation levels in the CpG sites of the PIGR gene on the prognosis(OS) of BRCA patients.

CpG island	HR (CI)	LR test p-value
PIGR-TSS1500-Open_Sea-cg00961792	0.787 (0.512;1.209)	0.28
PIGR-TSS1500-Open_Sea-cg01965508	1.223 (0.757;1.978)	0.40
PIGR-5’UTR;1stExon-Open_Sea-cg02105856	1.249 (0.846;1.845)	0.26
PIGR-3’UTR-Open_Sea-cg09763644	1.234 (0.768;1.982)	0.38
PIGR-TSS200-Open_Sea-cg12751565	0.644 (0.433;0.957)	0.027
PIGR-5’UTR-Open_Sea-cg15928480	0.583 (0.391;0.869)	0.0071
PIGR-Body-Open_Sea-cg17453671	1.43 (0.868;2.354)	0.15
PIGR-TSS1500-Open_Sea-cg20953047	0.801 (0.535;1.199)	0.29

CI, confidence interval; LR, likelihood ratio; HR, hazard ratio.

### PIGR differentially expressed genes are mainly involved in the immune response

**Figure 5:**
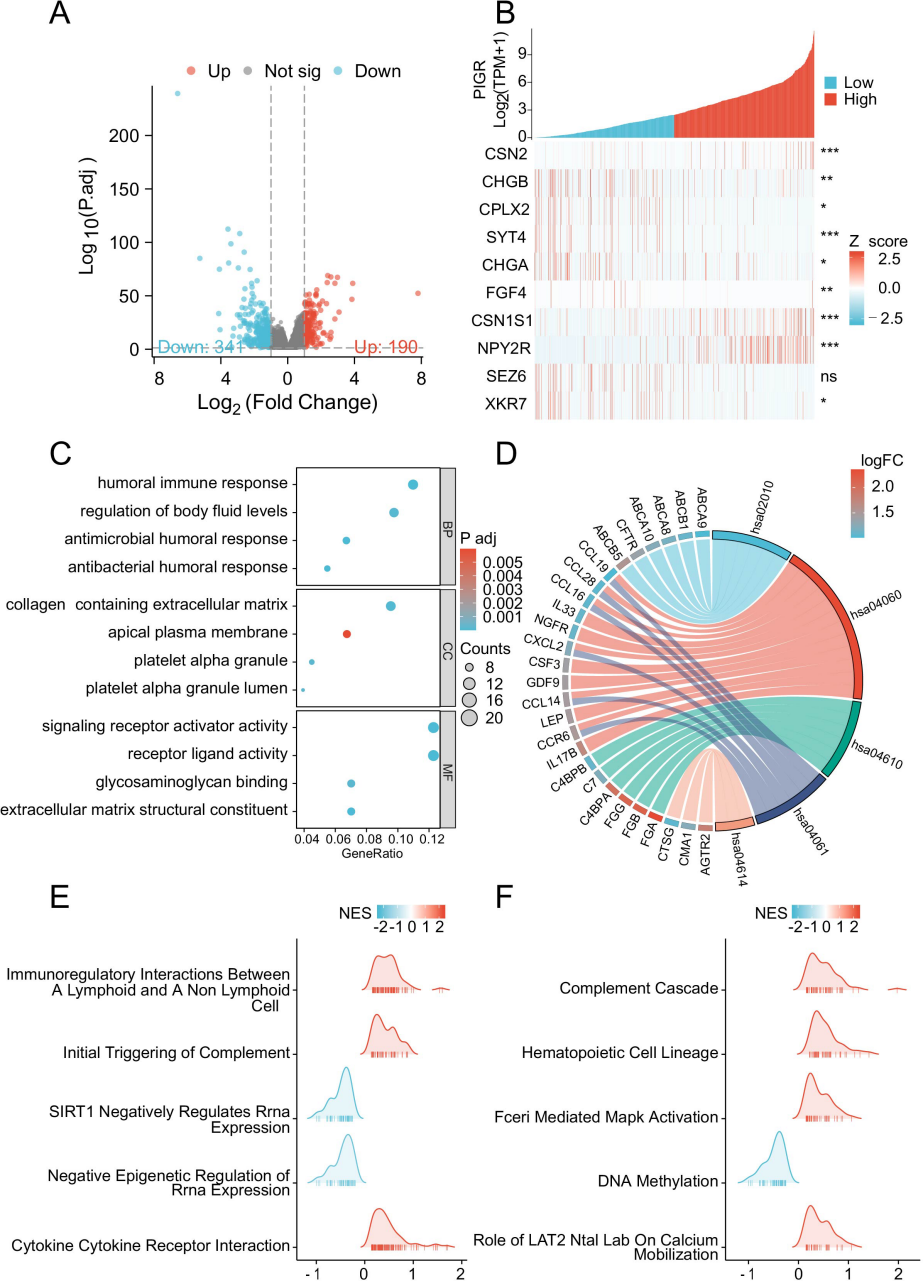
PIGR-associated differentially expressed genes (DEGs) and functional enrichment analysis via GO, KEGG and GSEA. **(A)** Volcano Plot of DEGs, with Red Indicating Significantly Upregulated DEGs and Blue Indicating Significantly Downregulated DEGs. **(B)** Heatmap of the Top Ten DEGs Correlated with PIGR Expression. **(C)** GO Analysis of DEGs. **(D)** KEGG Analysis of DEGs. **(E, F)** GSEA of DEGs. (DEGs, differentially Expressed Genes; GO, gene ontology; KEGG, Kyoto encyclopedia of genes and genomes; GSEA, gene set enrichment analysis.).

To elucidate the potential mechanisms underlying the inhibitory effects of PIGR on breast cancer proliferation, we leveraged the TCGA database to identify DEGs associated with PIGR expression. Specifically, we compared gene expression profiles between high and low PIGR expression groups in breast cancer patients and identified a total of 531 DEGs.This cohort included 190 up-regulated DEGs (35.8%) and 341 down-regulated DEGs (64.2%) (**Figure 5A**). The relationship between the top 10 differentially expressed genes (CSN2, CHGB, CPLX2, SYT4, CHG4, FIF4, CSN1S1, NPY2R, SEZ6, and XKR7) and PIGR was shown in the form of heat maps (**Figure 5B**).

Subsequently, to uncover the potential functions of PIGR-associated DEGs, we performed GO enrichment analyses. This analysis categorized the DEGs into three main categories: biological processes, cellular components, and molecular functions, revealing significant enrichment within various GO terms. These included humoral immune response, regulation of body fluid levels, endocrine processes, collagen-containing extracellular matrix, receptor ligand activity, signaling receptor activator activity, glycosaminoglycan binding, and ABC-type transporter activity (**Figure 5C**).

Furthermore, KEGG pathway analysis indicated significant enrichment of DEGs in pathways such as cytokine-cytokine receptor interaction, complement and coagulation cascades, viral protein interaction with cytokine and cytokine receptor, and ABC transporters (**Figure 5D**).

Subsequently, GSEA was conducted to compare the high and low PIGR expression groups. The analysis revealed significant enrichment of immune-related biological processes in the high PIGR expression group. In contrast, pathways associated with immunosuppression were predominantly enriched in the low PIGR expression group. These findings suggest that elevated PIGR expression is correlated with an enhanced immune phenotype in breast cancer (**Figure 5E, F**).

### Positive correlation between PIGR expression and abundance of immune cell and immune-related molecules

**Figure 6:**
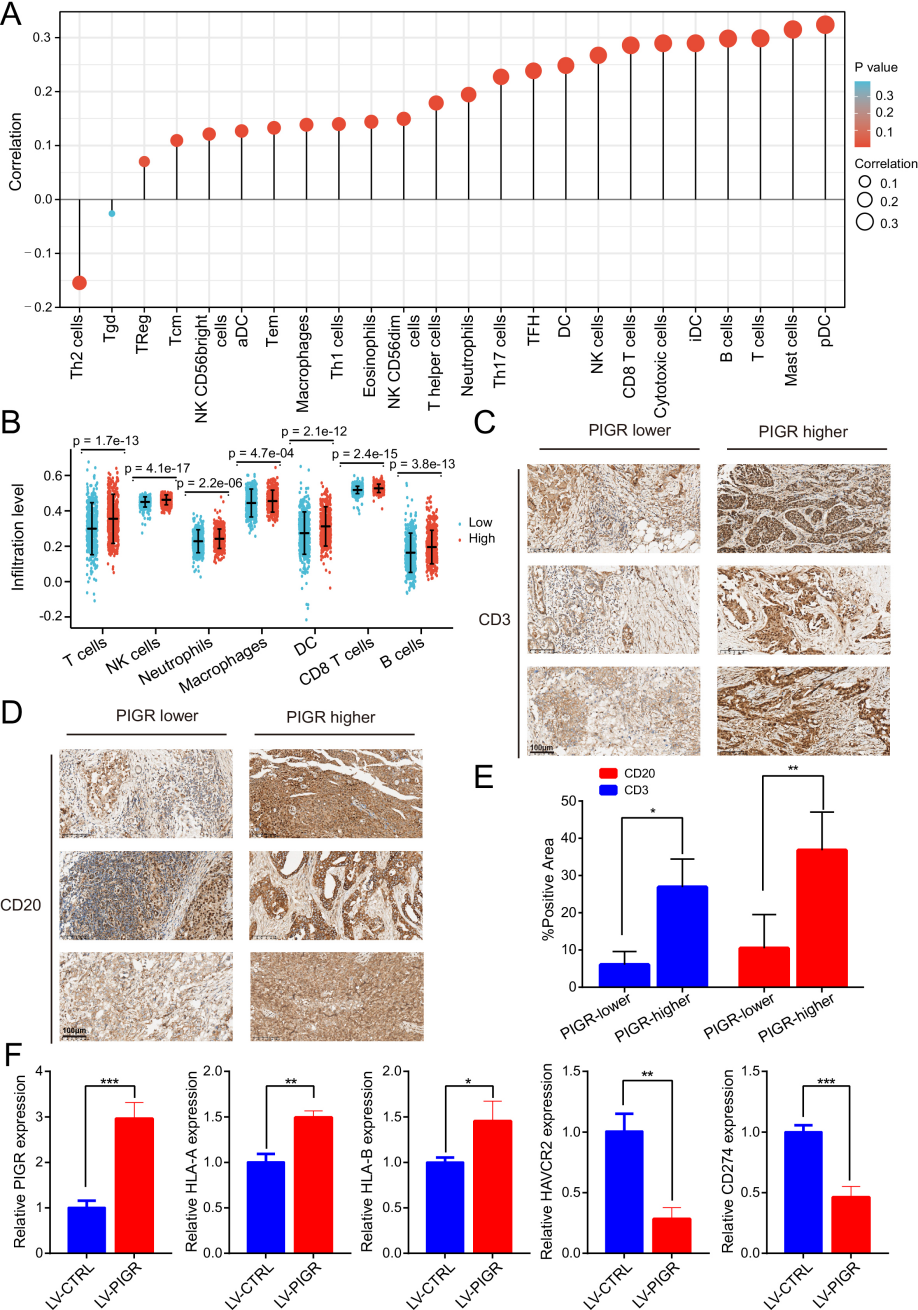
The expression level of PIGR was associated with the immune cell infiltration level in BRCA. **(A)** The correlation between PIGR expression level and the relative abundances of 24 immune cells. The color and size of the dots represent the P value and Spearman correlation coefficient, respectively. **(B)** Differential infiltration levels of 7 primary immune cells in PIGR-high and -low expression groups showed in scatter plots. **(C)** Evaluate the T lymphocyte marker CD3 expression levels in breast cancer tissues with relatively low and high PIGR expression by immunohistochemical. **(D)** Evaluate the B lymphocyte marker CD20 expression levels in breast cancer tissues with relatively low and high PIGR expression by immunohistochemical. **(E)** The proportion of CD3 and CD20 positive areas in the PIGR relatively low expression group and the PIGR relatively high expression group. **(F)** Relative expression of PIGR, HLA-A, HLA-B, CD274, and HAVCR2 in PIGR-Overexpressing and Control MCF-7 breast cancer cells.

To explore the relationship between changes in PIGR expression and the tumor microenvironment, we conducted immune cell infiltration analysis and immune-related signatures analysis. The result showed that the infiltration levels of most (22/24) immune cell types were significantly positively correlated with PIGR expression levels (**Figure 6A**). The infiltration levels of seven typical immune cell types -T cells, NK cells, Neutrophils, Macrophages, Dendritic cells, CD8 T cells, and B cells-were significantly higher in the high-PIGR expression groups than in the low-PIGR expression group (**Figure 6B**). Additionally, there was a significantly positive correlation between PIGR expression and the infiltration levels of these cell types (**Supplementary Figure 5**). We further confirmed this association by examining the expression of CD3 (**Figure 6C**) and CD20 (**Figure 6D**) in tumor tissues with low and high PIGR expression. Further analysis revealed that most immune-related molecules examined (TILs, immune inhibitors, immunostimulators, MHC molecules, chemokines, and receptors) were positively correlated with PIGR expression in BRCA (**Supplementary Figure 6**). The top six PIGR-positive related molecules in the BRCA cohort were shown in correlation scatter plots (**Supplementary Figure 7**, **8**).

To investigate whether the PIGR affects the expression of genes encoding some key immunomodulatory molecules, we extracted RNA from MCF-7 breast cancer cell lines with PIGR overexpression and control cell lines, and detected the expression of these genes by qPCR. The expression levels of *HLA-A* and *HLA-B*, which encode HLA class I molecules, were higher in the PIGR-overexpressing group than in the control group. In contrast, the expression levels of *CD274* (encoding PD-L1) and *HAVCR2* (encoding TIM-3) were lower in the PIGR-overexpressing group than in the control group (**Figure 6F**). These results demonstrated that PIGR might enhance tumor immunity in BRCA.

## Discussion

Breast cancer is a heterogeneous disease. Current prognostic indicators used in the clinic have certain limitations. Therefore, selecting a new clinicopathological indicator is necessary to predict the prognosis of BRCA patients. The PIGR is one of the most vital components of mucosal immunity, playing a pivotal role in mediating the transcytosis of polymeric immunoglobulins on epithelial surfaces to protect against invading pathogens (35). Research has revealed that PIGR exerted paradoxical effects on human tumors through complex mechanisms. For instance, PIGR can promote the tumorigenesis and metastasis of hepatocellular carcinoma (HCC) (13–15, 36). It may also be involved in the progression of pancreatic ductal adenocarcinoma (PDAC) and serve as a poor prognostic biomarker for PDAC (16, 17). Nevertheless, PIGR has been shown to play an anti-tumor role in several other human malignancies. PIGR expression is significantly downregulated and associated with favorable prognosis in nasopharyngeal carcinoma (NPC) and colorectal cancer (18, 37, 38). Additionally, PIGR plays an anti-tumor role in ESCC tumor immunity in an antigen-specific manner during NY-ESO-1 vaccinations (19). Loss of PIGR expression contributes to lung tumorigenesis, and PIGR may suppress lung tumorigenesis by maintaining cell differentiation (20). High PIGR expression independently predicts a decreased risk of recurrence and improved survival in patients with adenocarcinoma of the upper gastrointestinal tract (21). Similarly, high tumor-specific expression of PIGR is associated with a favorable prognosis in epithelial ovarian cancer (EOC) (22).

Our bioinformatic analysis and IHC staining showed that PIGR was significantly low-expressed in BRCA tissues. Down-regulated expression of PIGR had no significant association with proliferation and clone formation but significantly increased the migration of MCF-7 breast cancer cells. Conversely, overexpression of PIGR not only inhibited the proliferation and cloning of breast cancer cells but also suppressed their migration. *In vivo* experiments further revealed that overexpression of PIGR inhibits tumor growth. These findings suggest that PIGR might play an anti-tumor role in breast cancer cells. Based on data from the Kaplan-Meier plotter and the GSE86166 dataset, we found that PIGR expression is correlated with favorable prognosis in BRCA patients, as indicated by improved OS and RFS. Multivariate Cox regression analysis demonstrated that PIGR acted as an independent prognostic protective biomarker in BRCA. PIGR has particular value in predicting the prognosis of BRCA patients over a relatively long time scale (5 years). However, it still has room for improvement compared with tools such as PREDICT4 (39), Oncotype DX (40), MammaPrint (41), and EndoPredict (42). In the future, We plan to explore the molecular mechanism of PIGR and other breast cancer biomarkers and integrate them to improve prediction accuracy. This approach may provide clinicians with a novel method to forecast the long-term outcomes of BRCA patients.

The potential of leveraging the innate immune system to combat various malignancies has significantly broadened the horizons of cancer treatment. In our study, we identified that the differentially expressed genes of PIGR are predominantly enriched within immune-related annotations and pathways associated with breast cancer (BRCA). Notably, PIGR exhibited a significant correlation with the abundance of various immune cells and immune-related molecules in BRCA. Prior research has established the pivotal role of T cells as the primary immune effector cells responsible for tumor cell cytotoxicity (43), with CAR-T cell therapy being a notable advancement approved for the treatment of B-cell malignancies. Furthermore, prior studies have demonstrated that the infiltration of CD8^+^ T cells within the tumor microenvironment (TME) of lung adenocarcinoma (LUAD) is critical for eliciting effective anti-tumor immune responses (44). Other immune cell types, including neutrophils, contribute to anti-tumor immunity through the activation of immune responses targeting tumor cells and through direct lysis of malignant cells (45). Dendritic cells also play a crucial role by activating CD8^+^ T cells, thereby initiating anti-tumor immunity (46). Additionally, tumor-infiltrating lymphocytes (TILs) serve as an anti-tumor immune response indicator and are associated with a better prognosis in breast cancer (47, 48). Building on this foundational research in immunotherapy, numerous immune modulators that trigger immune responses have been developed and are being applied in cancer immunotherapy (49). Immune cells are guided into the tumor through interactions between chemokines and their receptors, and chemokine receptors expressed in tumor and immune cells are strongly associated with patient prognosis (50).

Specifically, in rectal cancer, PIGR expression has been associated with the expression of several immune checkpoints, including IDO1, CD274, PDCD1, CTLA4, and LAG3 (37). Moreover, IgA is the most abundant antibody produced by the human body, and most IgA exists as dimers at mucosal sites. The ability of dimeric IgA (dIgA) to bind to PIGR is critical for its transport across the mucosal epithelial barrier and its transformation into secretory IgA (sIgA) (51). IgA can be transported into the tumor microenvironment via PIGR-mediated mechanisms (52), where it specifically recognizes and expels mutated oncogenic proteins in cells, thereby inhibiting tumor growth (53, 54). This process may also modulate immune cell activity within the tumor milieu. IgA may influence the polarization state of immune cells, including macrophages and T cells, thereby impacting tumor progression and immune evasion (55, 56). Additionally, the targeted delivery of small molecules and gene therapeutic agents to mucosal epithelial cells via the PIGR-mediated pathway (57) could increase local drug concentrations and reduce systemic side effects, which provides a broad application prospect for PIGR in mediating targeted therapy.

Given the significant positive correlation between PIGR and the abundance of these immune cells in BRCA, we postulate that PIGR may regulate the distribution and activity of immune cells through the modulation of immune checkpoints and the transportation of IgA. In this study, GSEA revealed that PIGR differentially expressed genes mainly focused on immune-related annotations and pathways in BRCA. PIGR was significantly correlated with the abundance of various immune cells and immune-related molecules in BRCA. Breast cancer cells can downregulate the expression of HLA class I molecules, thereby reducing the ability of CD8^+^T cells to recognize and kill tumor cells (58). At the same time, breast cancer cells highly express a variety of immunosuppressive molecules, such as Programmed Death Ligand 1 (PD-L1) and T-cell immunoglobulin and Mucin Domain 3 (TIM-3), which inhibit the activity of T cells (59–61). Our results suggest that PIGR overexpression may enhance the ability of breast cancer cells to present antigens to CD8^+^T cells, potentially counteracting the immune evasion mechanism of HLA class I downregulation. PIGR overexpression can downregulate the expression of PD-L1 and TIM-3, thereby reducing the inhibitory effects on T cell activity. These findings are significant as they suggest a potential role for PIGR in modulating the immune microenvironment of breast cancer cells. They indicate that PIGR might play an anti-tumor role by activating tumor immunity in BRCA tissues, thereby improving the prognosis of BRCA patients.

In conclusion, our study has conducted a comprehensive preliminary exploration of the relationship between PIGR and BRCA through bioinformatics analysis and both *in vitro* and *in vivo* experiments. We observed that high expression of PIGR in BRCA tissues is correlated with a favorable prognosis. We also explored the impact of PIGR expression on tumor cell behavior and preliminarily verified that this impact is associated with tumor immunity. Further in-depth investigation into the role of PIGR in tumor immunity is expected to provide a more comprehensive understanding of the mechanisms by which PIGR affects the development of breast cancer.

## Data Availability

The original contributions presented in the study are included in the article/**Supplementary Material**. Further inquiries can be directed to the corresponding authors.

## Supplementary material

Supplementary Figure 1 Inhibition of PIGR overexpression on MDA-MB-231 and SKBR3 breast cancer cells. **(A)** Western blotting was used to test the expression of PIGR in PIGR overexpressing MDA-MB-231 and SKBR3 cells. **(B)** Effects of PIGR on MDA-MB-231 and SKBR3 cell proliferation checked via the CCK-8 assay. **(C)** Effects of PIGR on MDA-MB-231 and SKBR3 cell clone formation checked via a colony formation assay. **(D)** Effects of PIGR on MDA-MB-231 and SKBR3 migration checked via the Transwell migration assay. (Abbreviations: OE-CTRL: cells transfected with empty vectors as control; OE-PIGR: cells transfected with plasmid to overexpressing PIGR).

Supplementary Figure 2 Influence of PIGR Downregulation on MCF-7 Cell Proliferation, Colony Formation, and Migration. **(A)** Western blotting was used to test the down-regulated expression of PIGR in PIGR knockdown MCF-7 cells. (**B)** Effects of PIGR on MCF-7 cell proliferation checked via the CCK-8 assay. (**C)** Effects of PIGR on MCF-7 cell clone formation checked via a colony formation assay. (**D)** Effects of PIGR on MCF-7 migration checked via the Transwell migration assay. (Abbreviations: shCTRL, MCF-7 cells transfected with a short hairpin RNA as control; shPIGR, MCF-7 cells transfected with a short hairpin RNA to knockdown PIGR).

Supplementary Figure 3 DNA promoter methylation levels of the PIGR gene in BRCA. **(A)** Differential promoter methylation levels of PIGR in BRCA and normal breast tissues were obtained from the UALCAN. **(B)** The methylation levels of CpG islands in the PIGR gene in BRCA were obtained from the MethSurv.

Supplementary Figure 4 Nomogram and calibration curves to predict BRCA patients’ 1-, 3-, and 5-year OS probability. **(A)** Nomogram for predicting BRCA patients’ likelihood of 1-, 3-, and 5-year OS. (**B)** Calibration curves of the nomogram prediction of 1-year OS of BRCA patients. **(C)** Calibration curves of the nomogram prediction of 3-year OS of BRCA patients. **(D)** Calibration curves of the nomogram prediction of 5-year OS of BRCA patients.

Supplementary Figure 5 Correlation between PIGR and immune infiltration levels of 7 typical immune cells. **(A)** The correlations between PIGR expression and the infiltration levels of T cells. **(B)** The correlations between PIGR expression and the infiltration levels of NK (natural killer) cells. **(C)** The correlations between PIGR expression and the infiltration levels of Neutrophils. **(D)** The correlations between PIGR expression and the infiltration levels of Macrophages. **(E)** The correlations between PIGR expression and the infiltration levels of Dendritic cells. **(F)** The correlations between PIGR expression and the infiltration levels of CD8 T cells. **(G)** The correlations between PIGR expression and the infiltration levels of B cells.

Supplementary Figure 6 Associations between PIGR expression and an abundance of TILs, immunomodulators, chemokines, and receptors in pan-cancer from the TISIDB database. **(A)** Associations between PIGR expression and an abundance of TILs. (**B-D)** Associations between PIGR expression and an abundance of immunomodulators including immunoinhibitors, immunostimulators, and MHC molecules. (**E-F)** Associations between PIGR expression and an abundance of chemokines and receptors.

Supplementary Figure 7 Top six PIGR-associated TILs, immunoinhibitors and immunostimulators in the BRCA cohor**t. (A-F)** Top six PIGR-associated TILs in the BRCA cohort. **(G-L)** Top six PIGR-associated immunoinhibitors in the BRCA cohort. **(M-R)** Top six PIGR-associated immunostimulators in the BRCA cohort.

Supplementary Figure 8 Top six PIGR-associated MHC molecules, chemokines and receptors in the BRCA cohor**t. (A-F)** Top six PIGR-associated MHC molecules in the BRCA cohort. **(G-L)** Top six PIGR-associated chemokines in the BRCA cohort. **(M-R)** Top six PIGR-associated receptors in the BRCA cohort.
