# Supplementary figures and images for: PIGR predicts good clinical outcomes and plays a tumor suppressor role in the development of breast cancer
Source: Front Oncol. 2025 May 2;15:1439120. doi: 10.3389/fonc.2025.1439120 (PMC12081344; doi:10.3389/fonc.2025.1439120)

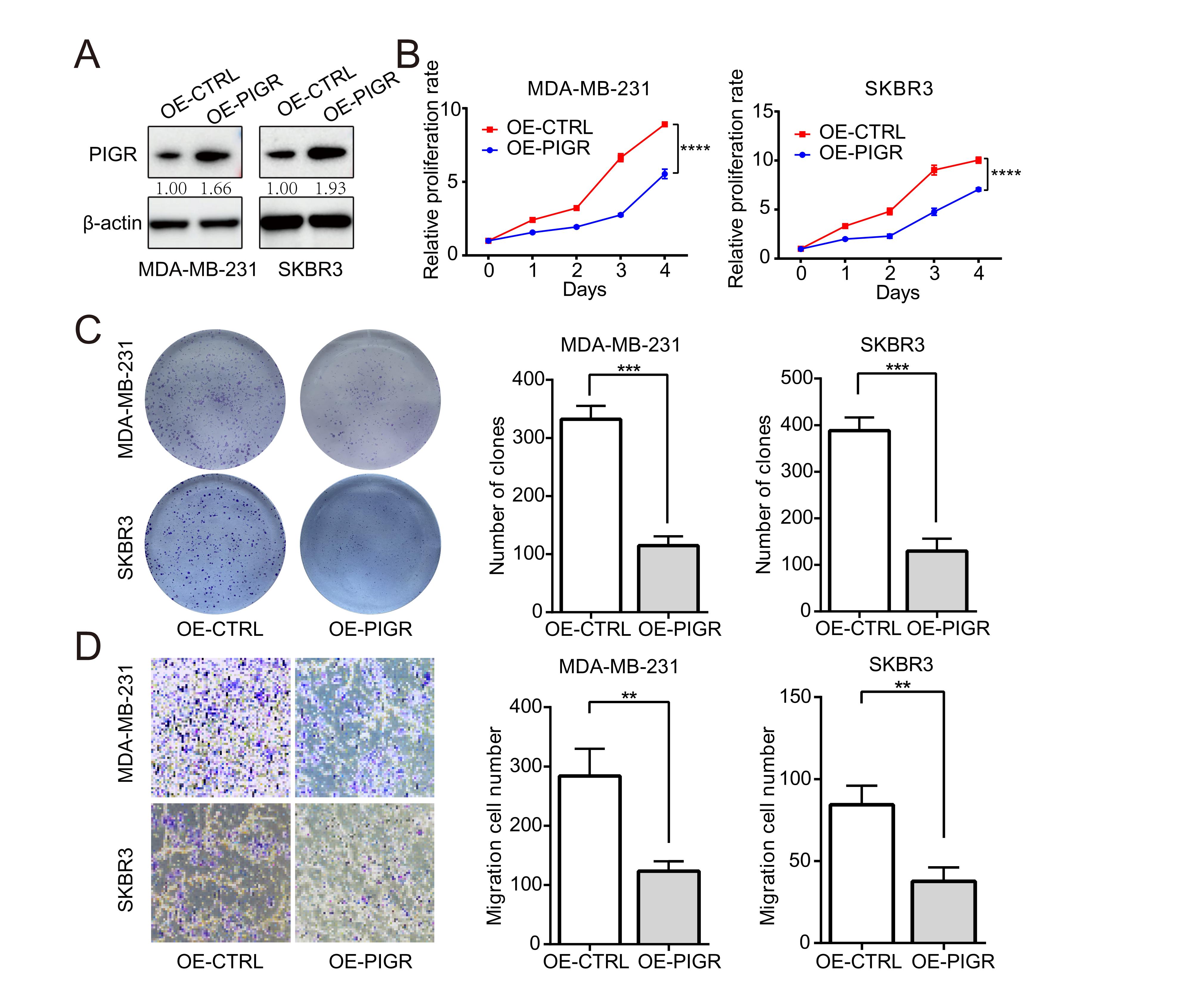

Supplement: Supplementary Figure 1 — Inhibition of PIGR overexpression on MDA-MB-231 and SKBR3 breast cancer cells. (A) Western blotting was used to test the expression of PIGR in PIGR overexpressing MDA-MB-231 and SKBR3 cells. (B) Effects of PIGR on MDA-MB-231 and SKBR3 cell proliferation checked via the CCK-8 assay. (C) Effects of PIGR on MDA-MB-231 and SKBR3 cell clone formation checked via a colony formation assay. (D) Effects of PIGR on MDA-MB-231 and SKBR3 migration checked via the Transwell migration assay. (Abbreviations: OE-CTRL: cells transfected with empty vectors as control; OE-PIGR: cells transfected with plasmid to overexpressing PIGR). [file Image1.jpeg]

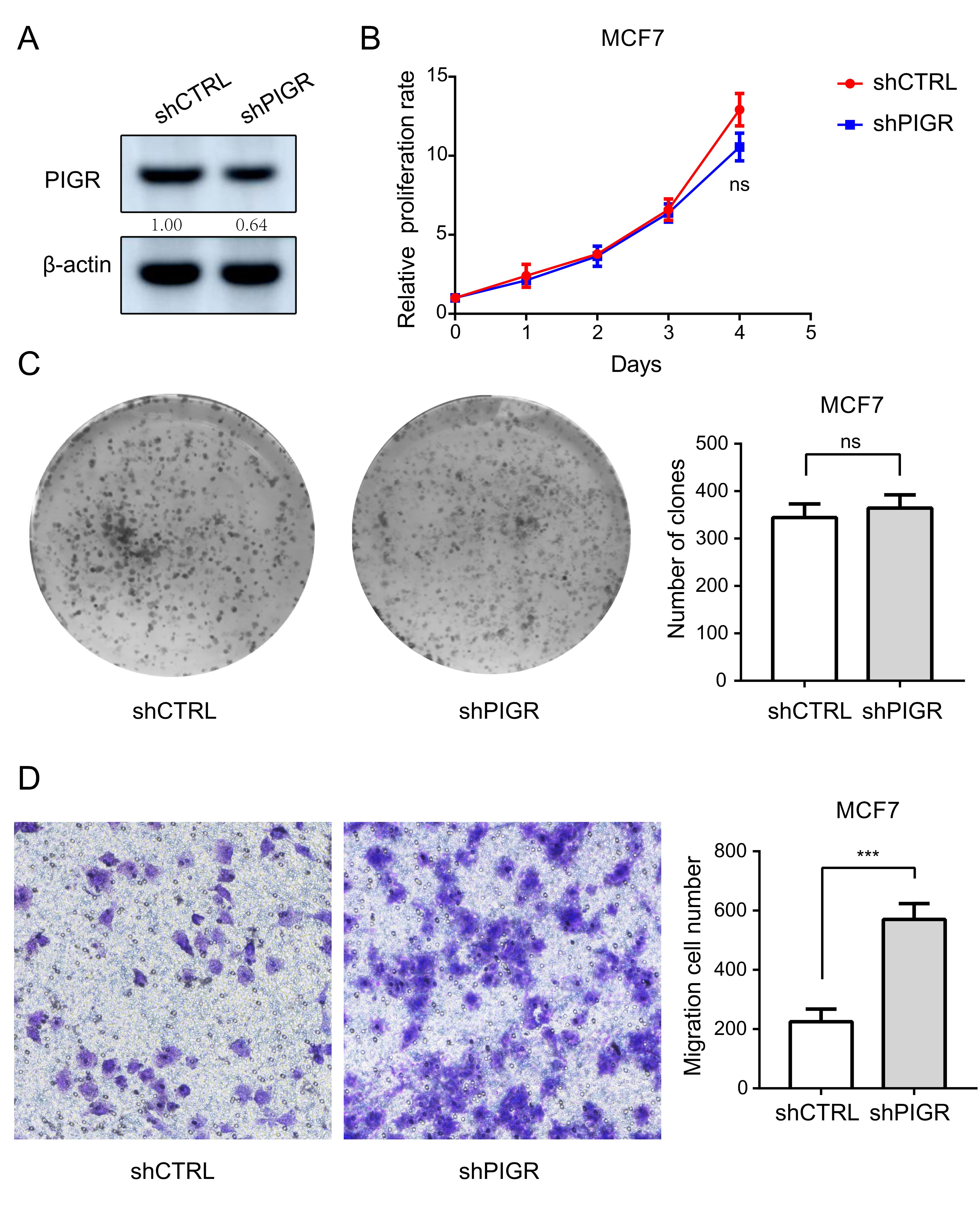

Supplement: Supplementary Figure 2 — Influence of PIGR Downregulation on MCF-7 Cell Proliferation, Colony Formation, and Migration. (A) Western blotting was used to test the down-regulated expression of PIGR in PIGR knockdown MCF-7 cells. (B) Effects of PIGR on MCF-7 cell proliferation checked via the CCK-8 assay. (C) Effects of PIGR on MCF-7 cell clone formation checked via a colony formation assay. (D) Effects of PIGR on MCF-7 migration checked via the Transwell migration assay. (Abbreviations: shCTRL, MCF-7 cells transfected with a short hairpin RNA as control; shPIGR, MCF-7 cells transfected with a short hairpin RNA to knockdown PIGR). [file Image2.jpeg]

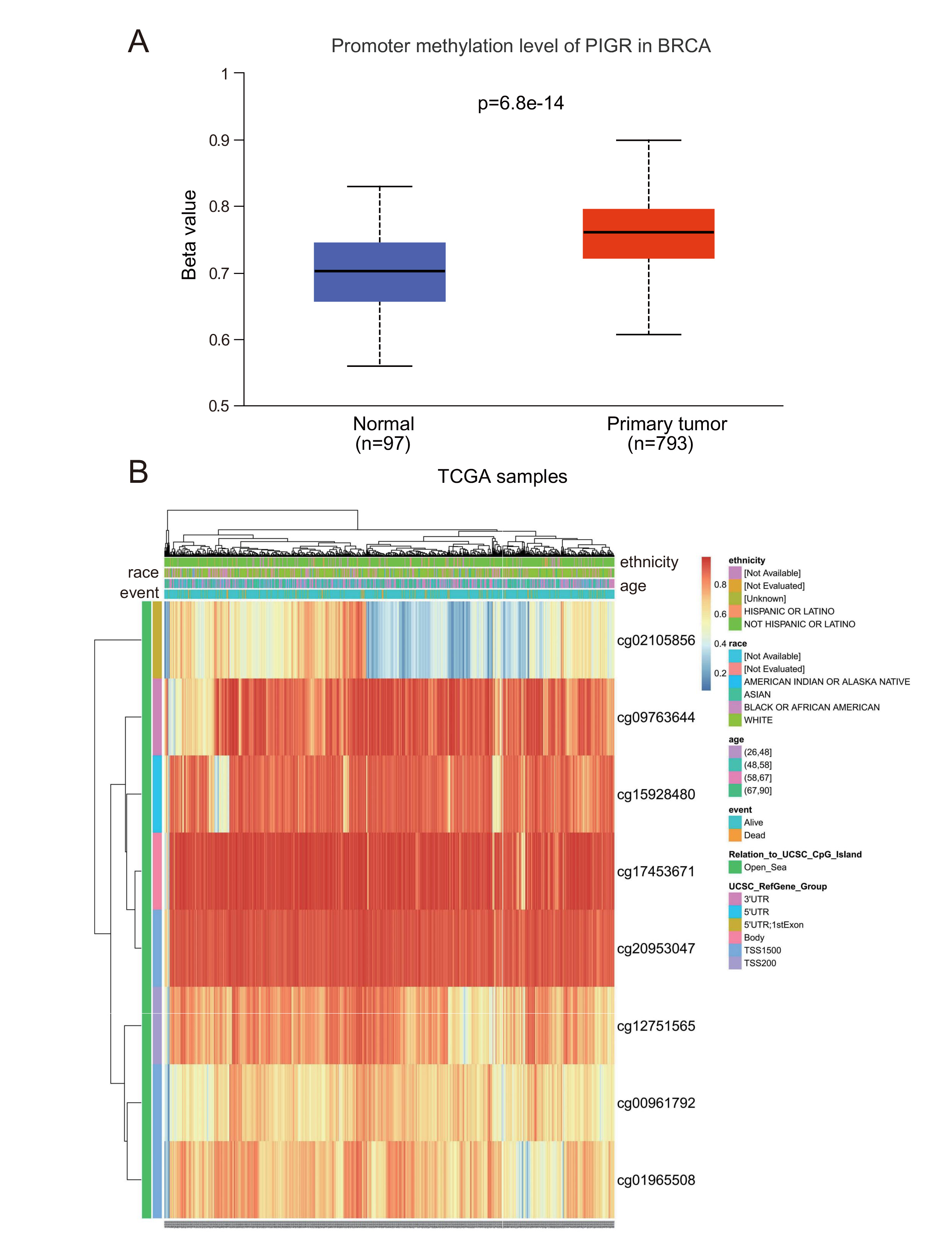

Supplement: Supplementary Figure 3 — DNA promoter methylation levels of the PIGR gene in BRCA. (A) Differential promoter methylation levels of PIGR in BRCA and normal breast tissues were obtained from the UALCAN. (B) The methylation levels of CpG islands in the PIGR gene in BRCA were obtained from the MethSurv. [file Image3.jpeg]

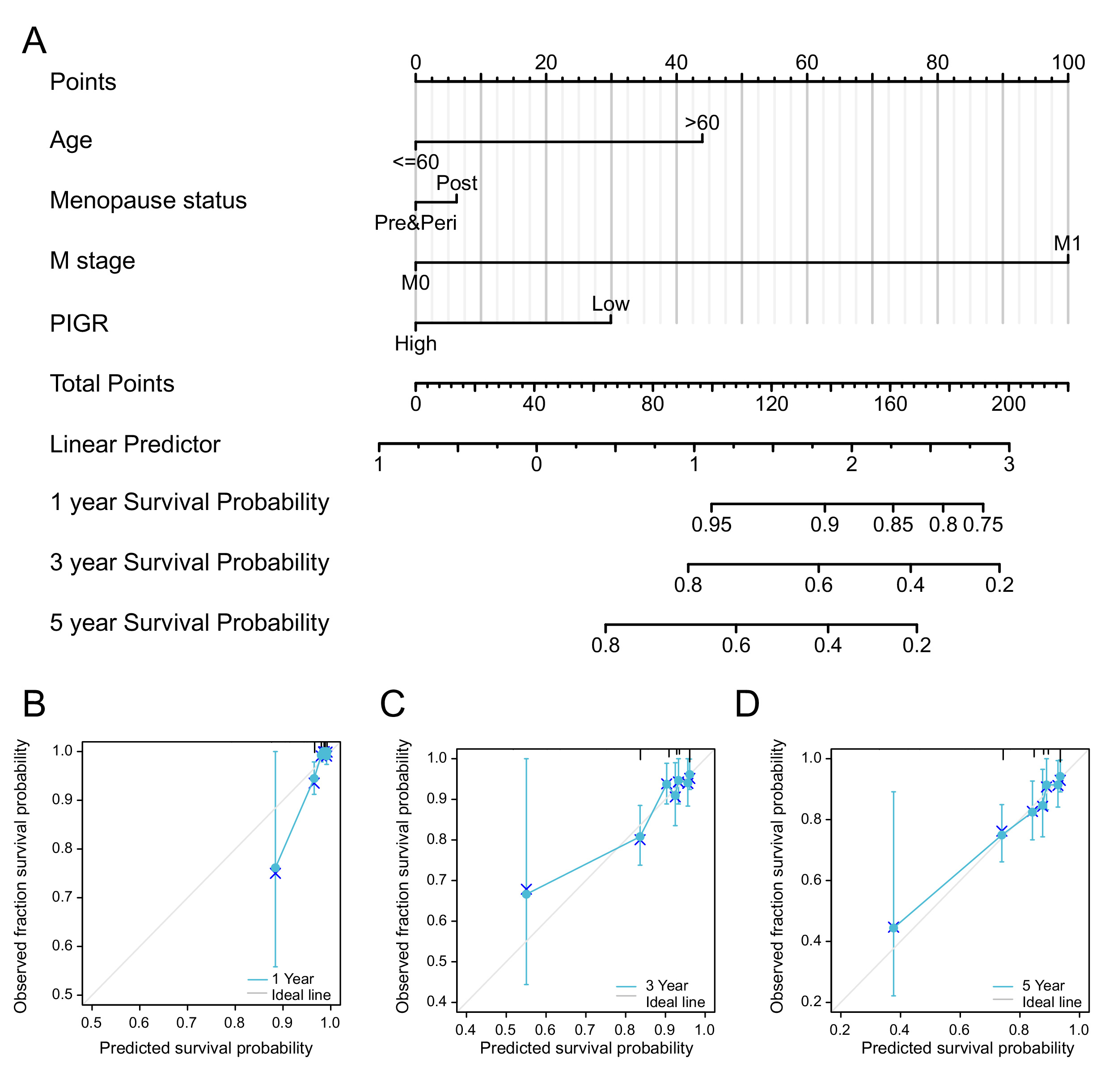

Supplement: Supplementary Figure 4 — Nomogram and calibration curves to predict BRCA patients’ 1-, 3-, and 5-year OS probability. (A) Nomogram for predicting BRCA patients’ likelihood of 1-, 3-, and 5-year OS. (B) Calibration curves of the nomogram prediction of 1-year OS of BRCA patients. (C) Calibration curves of the nomogram prediction of 3-year OS of BRCA patients. (D) Calibration curves of the nomogram prediction of 5-year OS of BRCA patients. [file Image4.jpeg]

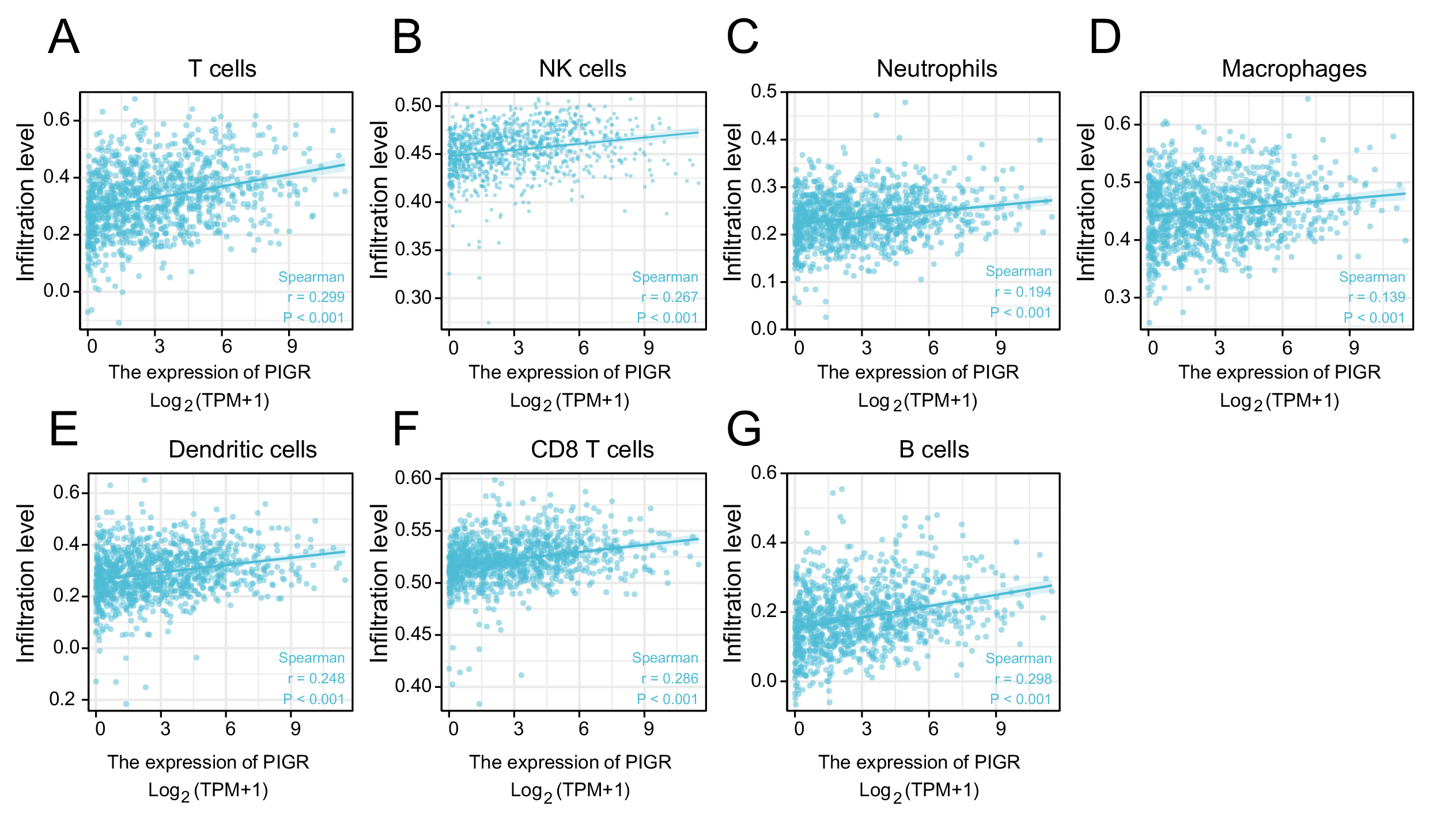

Supplement: Supplementary Figure 5 — Correlation between PIGR and immune infiltration levels of 7 typical immune cells. (A) The correlations between PIGR expression and the infiltration levels of T cells. (B) The correlations between PIGR expression and the infiltration levels of NK (natural killer) cells. (C) The correlations between PIGR expression and the infiltration levels of Neutrophils. (D) The correlations between PIGR expression and the infiltration levels of Macrophages. (E) The correlations between PIGR expression and the infiltration levels of Dendritic cells. (F) The correlations between PIGR expression and the infiltration levels of CD8 T cells. (G) The correlations between PIGR expression and the infiltration levels of B cells. [file Image5.jpeg]

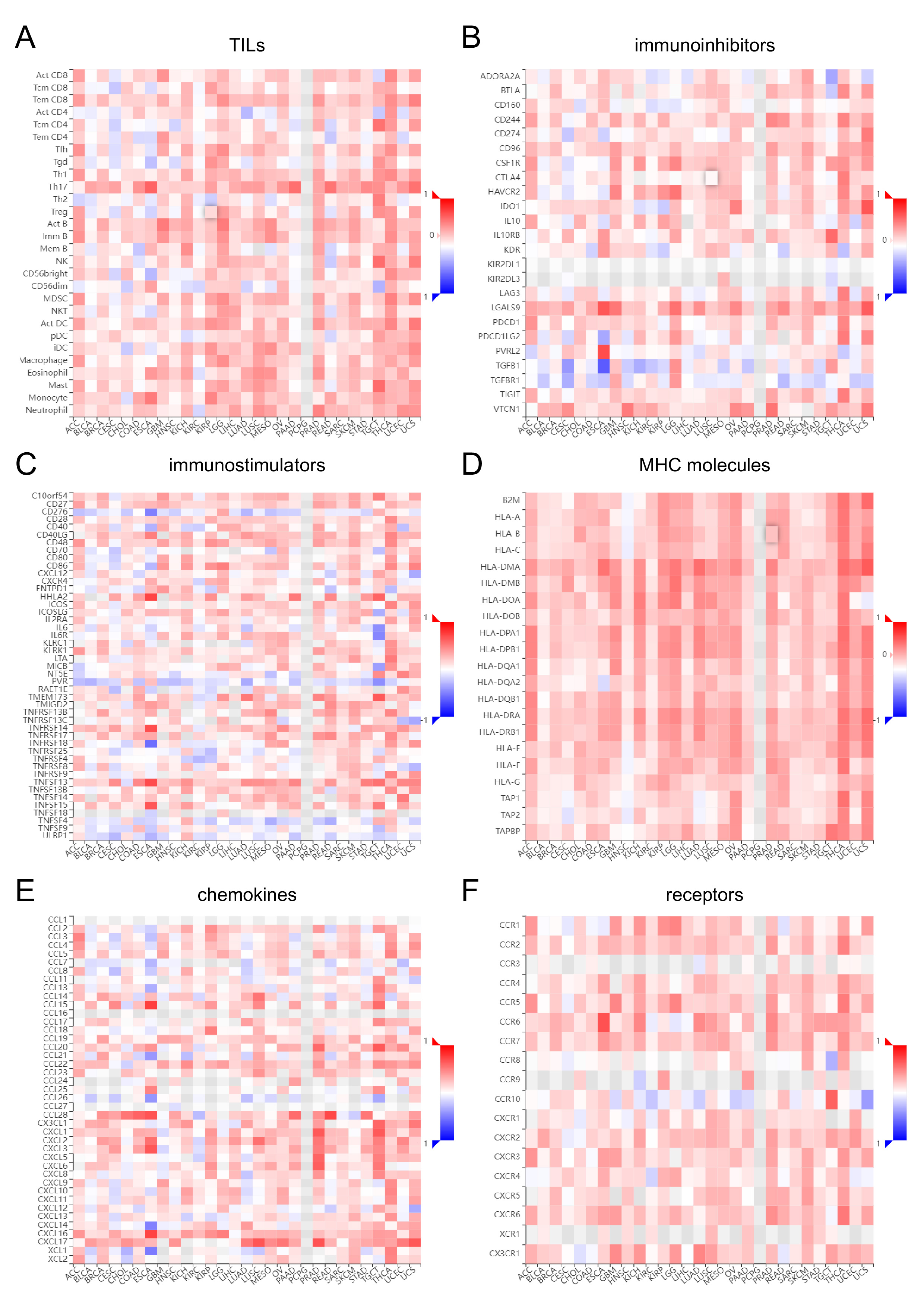

Supplement: Supplementary Figure 6 — Associations between PIGR expression and an abundance of TILs, immunomodulators, chemokines, and receptors in pan-cancer from the TISIDB database. (A) Associations between PIGR expression and an abundance of TILs. (B-D) Associations between PIGR expression and an abundance of immunomodulators including immunoinhibitors, immunostimulators, and MHC molecules. (E-F) Associations between PIGR expression and an abundance of chemokines and receptors. [file Image6.jpeg]

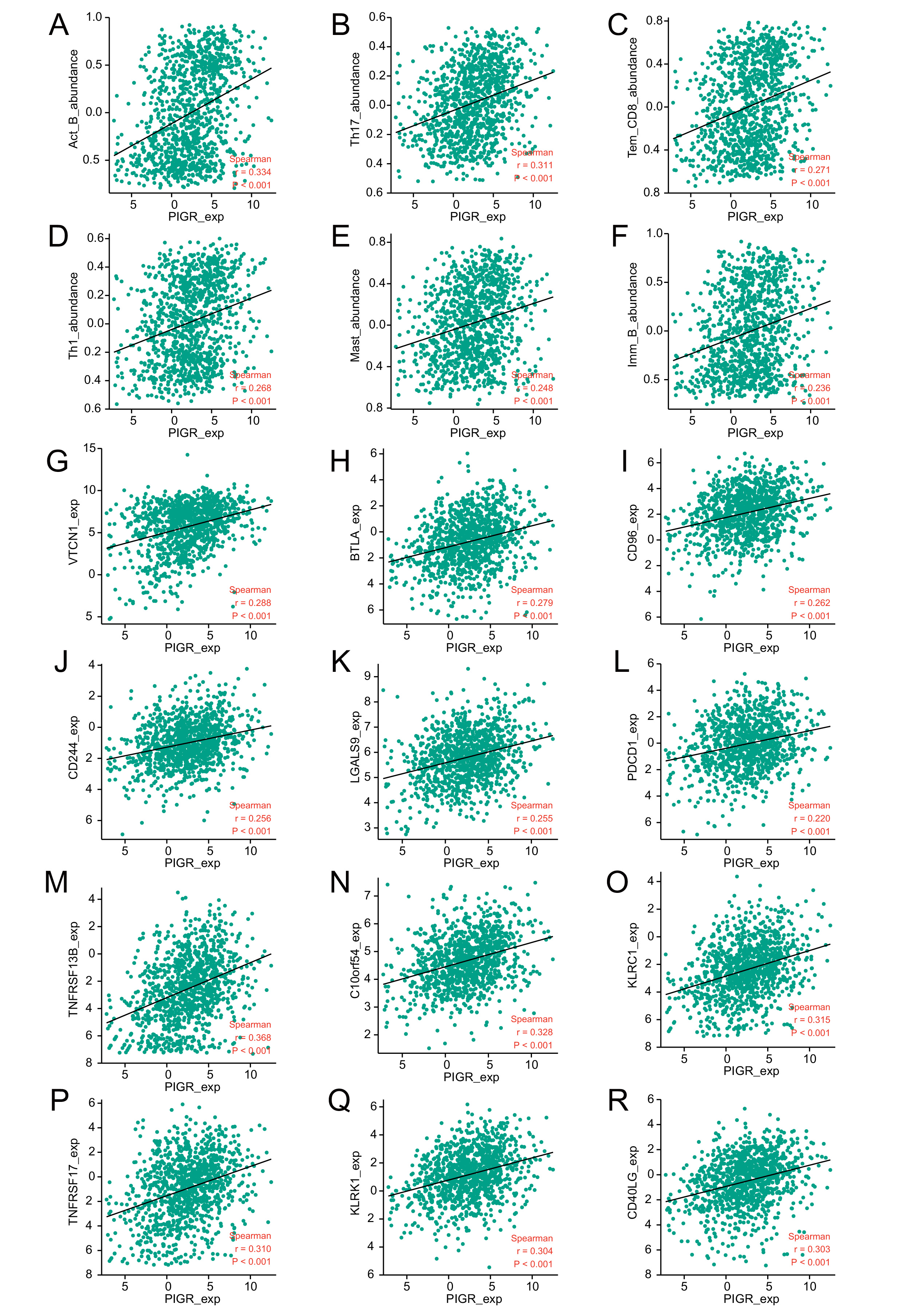

Supplement: Supplementary Figure 7 — Top six PIGR-associated TILs, immunoinhibitors and immunostimulators in the BRCA cohort. (A-F) Top six PIGR-associated TILs in the BRCA cohort. (G-L) Top six PIGR-associated immunoinhibitors in the BRCA cohort. (M-R) Top six PIGR-associated immunostimulators in the BRCA cohort. [file Image7.jpeg]

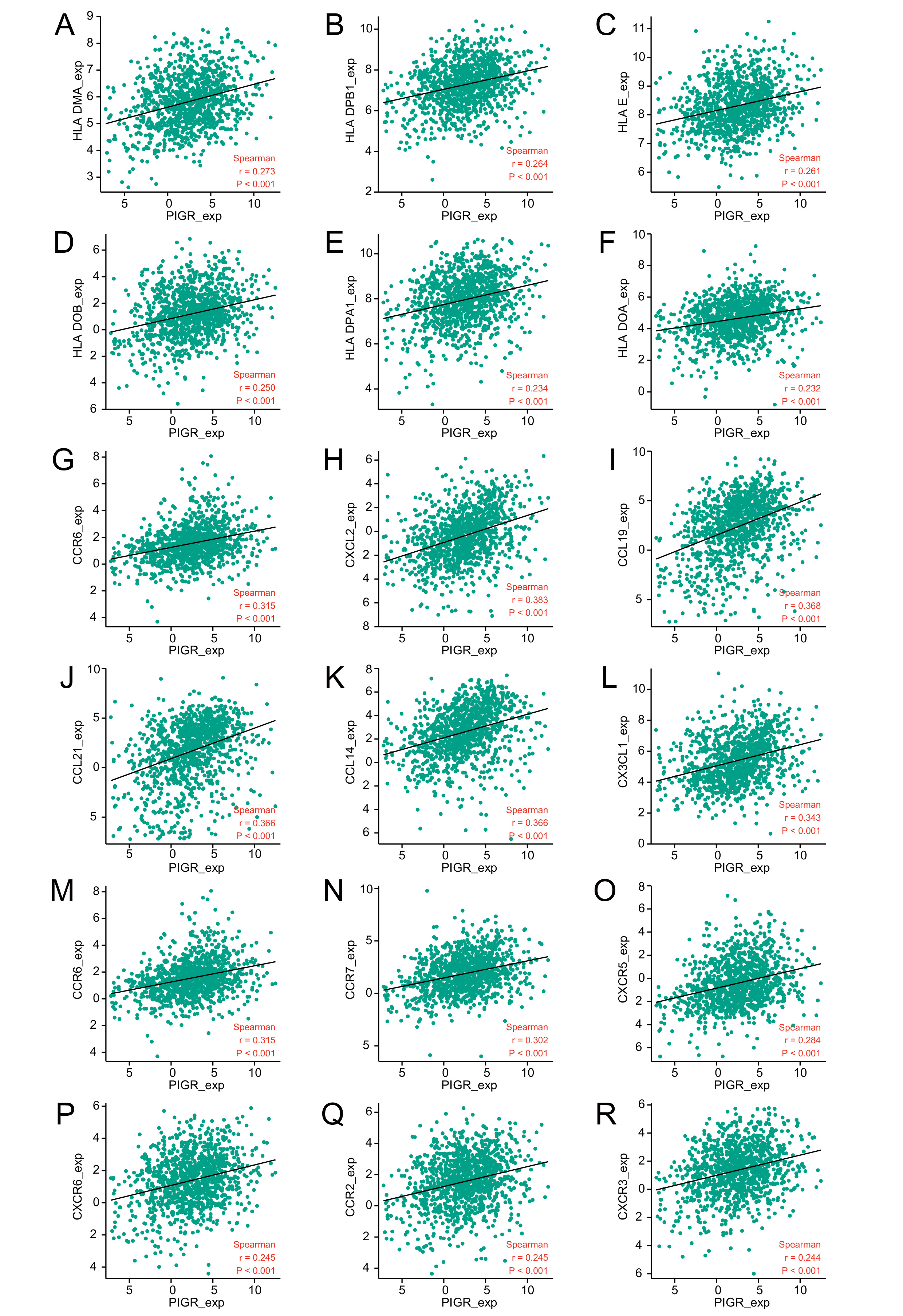

Supplement: Supplementary Figure 8 — Top six PIGR-associated MHC molecules, chemokines and receptors in the BRCA cohort. (A-F) Top six PIGR-associated MHC molecules in the BRCA cohort. (G-L) Top six PIGR-associated chemokines in the BRCA cohort. (M-R) Top six PIGR-associated receptors in the BRCA cohort. [file Image8.jpeg]
